# Medical management of a child treated for two unique envenomation episodes via captive snakes in a 60-day period: A case report

**DOI:** 10.1016/j.heliyon.2024.e40245

**Published:** 2024-11-07

**Authors:** Ming Gao, Xiangxing Zhang, Tianzi Jian, Cece Sun, Guangcai Yu, Yikai Gao, Baotian Kan, Xiangdong Jian

**Affiliations:** a Department of Poisoning and Occupational Diseases, Emergency Medicine, Qilu Hospital of Shandong University, Cheeloo College of Medicine, Shandong University, Jinan, Shandong, 250012, China; b Department of Emergency Medicine, Peking University Third Hospital, Beijing, 100191, China; c Department of Occupational and Environmental Health, School of Public Health, Cheeloo College of Medicine, Shandong University, Jinan, Shandong, 250012, China; d Department of Hematology, Qilu Hospital of Shandong University, Cheeloo College of Medicine, Shandong University, Jinan, Shandong, 250012, China; e Department of Nursing, Department of Gerontology, Qilu Hospital, Cheeloo College of Medicine, Shandong University, Jinan, Shandong, 250012, China

**Keywords:** Snake bite, *Bungarus multicinctus*, *Gloydius brevicaudus*, Antivenom, Necrosis

## Abstract

Venomous snake bites can result in irreversible damage, leading to respiratory dysfunction, bleeding disorders, kidney damage, or serious complications. In recent years, with the popularity of online shopping in China, snakes can be easily purchased and kept as pets, even if some areas are not natural habitats for certain kinds of snakes. A 13-year-old boy purchased two venomous snakes online as pets. On April 16, 2019,the boy was bitten by a *B. multicinctus(Bungarus multicinctus)*, and he had mild difficulty breathing and limb weakness. He was discharged after symptomatic treatment with B. multicinctus antivenom. On June 17, 2019, the boy was bitten a second time by a *short-tailed pit viper (Gloydius brevicaudus)* resulting in swelling and necrosis of the left hand. After a systemic and comprehensive treatment was implemented, including Agkistrodon halys antivenom injections, infection control, hormone therapy, improved circulation, negative pressure wound therapy, skin graft, and nutritional support, the boy recovered. This case provides valuable insights for diagnosing and treating venomous snake bites and their complications while also raising public awareness about the hazards of purchasing venomous snakes online,and it also provide case support for the improvement of online shopping for minors, wildlife protection, and live animal mailing management, helping to prevent such incidents from happening again.

## Highlights

• We report a case of a child who was bitten by his pet poisonous snakes.
• The patient was bitten first by *Bungarus multicinctus* and was treated successfully.
• He was then bitten two months later by a *Gloydius brevicaudus* and treated successfully.
• We discuss the dangers of purchasing unregulated venomous snakes online.

## 1 Introduction

Venomous snake bite poisoning is a serious and critical disease in clinical practice [1]. There are more than 3340 species of snakes worldwide, including more than 660 species of venomous snakes and nearly 200 species of deadly snakes. There are more than 1700 species of colubrid, more than 100 of which contain small amounts of toxins, and a few may be fatal. There are over 210 species of snakes in China, including 66 genera and nine families, with more than 60 species of venomous snakes and more than 10 species of highly venomous snakes [[2], [3], [4]]. Due to the wide latitude span between North and South China, the natural distribution of venomous snakes varies greatly. Venomous snakes are mainly distributed in southern China, including *Bungarus multicinctus (Bungarus)*, *cobras (Naja)* and *trimeresurus (Crotalinae)*. The venomous snakes in northern China are mainly *Gloydius*. Millions of snakebite cases occur every year, mostly during the peak farming season from June to July, of which 100,000 to 300,000 involve venomous snake bites, with a fatality rate of about 5 % [5]. Venomous snakebites cause 25–30 % of individuals to become disabled, and large numbers of people working in agriculture, forestry and fishing are at greater risk of snakebites [6], affecting the workforce and placing a heavy burden on families and society. Over the past decade, keeping venomous snakes as pets has gradually increased, and the Internet and online shopping offer great convenience to enthusiasts [7]. Although the Chinese government bans the mailing of live animals, it is impossible for express delivery companies to open and inspect the huge number of orders they receive daily. This study reports the case of a 13-year-old boy who purchased venomous snakes online as pets in April 16, 2019 and June 17, 2019. He was bitten by these snakes twice and suffered severe envenomation. He was brought to the Department of Poisoning and Occupational Diseases, Qilu Hospital of Shandong University, Shandong, China. After active treatment, the boy recovered and was discharged.

## 2 Case presentation

**Fig. 1:**
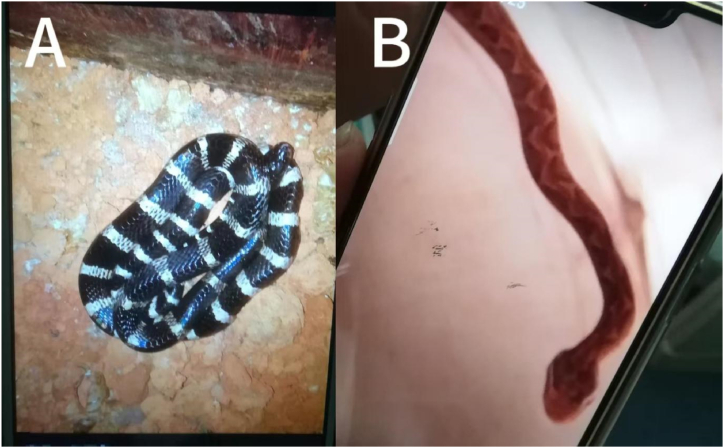
A, a *Bungarus multicinctus*; B,a *short-tailed pit vipers (Gloydius brevicaudus)*.

A boy aged 13 years bought a Bungarus multicinctus, also known as the many-banded krait or umbrella snake, online to keep as a pet and was bitten on the right hand on April 16, 2019. This snake's venom is neurotoxic. Half an hour later, he was brought to the local hospital. However, there was no *B. Multicinctus* antivenom in the local hospital. The patient's condition deteriorated and he had difficulty breathing. Four hours later, he was sent to the poisoning department of Shandong University Qilu Hospital. At admission, the boy complained of shortness of breath, dizziness, and difficulty lifting his upper limbs. Physical examination results were as follows: temperature, 37.1 °C; pulse rate, 78 beats/min; breathing rate, 18 beats/min; blood pressure, 122/76 mmHg; and SpO_2_, 98 %. Despite being conscious, he exhibited poor mental state and experienced general weakness, dizziness, and speech difficulties. Bilateral pupils were equally enlarged, homologous, and sensitive to light. Breathing was clear, with no dry and wet rales. Heart rate was 78 beats/min with a regular rhythm. The abdomen was flat and soft, with no tenderness and rebound pain. The liver and spleen were not palpable. There was no redness, swelling, or pain in the wound, only slight itching and numbness. Physiological reflexes were present, and pathological reflexes were not induced. The distal muscle strength of the upper extremity was level 5, and the proximal muscle strength was level 4. The upper limbs were weak, mainly on the affected side, and the limbs could lift slowly with shaking. The shoulder joints could resist partial resistance, and the elbow and wrist joints had normal movements and could grasp and bend. The muscle tone was normal. No abnormalities were detected in routine blood tests, biochemistry, and coagulation. The patient's father showed a photo of the snake that bit the boy, and it was confirmed as a small *B. multicinctus* (Fig. 1A). The admission diagnosis was *B. multicinctus* bite envenomation. Immediately after admission, the patient's blood oxygen saturation was monitored, and intravenous access was established. The patient was administered *B.multicinctus* antivenom. First, according to Chinese snakebite management consensus expert group (2018) [8] and the Instructions for *B. multicinctus* antivenom, a skin allergy test was performed, and the result was positive. Desensitization tests were then conducted according to the instruction, and 10,000 IU (10 ml) of *B. multicinctus* venom serum diluted 1:1 with normal saline was administered, a micro pump injection slowly administered dexamethasone 5 mg combined with a 250 ml saline drip. We prepared epinephrine and other rescue drugs and equipment to deal with adverse reactions. After the injection of antivenom, no allergic reaction or serum sickness type reactions occurred; electrolytes, liver and kidney function, and coagulation function were normal; The boy's symptoms gradually abated, his mood improved, absence of dizziness and fatigue, and his muscle strength returned to baseline. He was discharged after being hospitalized for five days. The chronological sequence of the first bites is shown in Table 1.

**Table 1:** The chronological sequence of the two bites.

chronological sequence	bite event 1	bite event 2
species	a Bungarus multicinctus	a short-tailed pit vipers (*Gloydius brevicaudus*)
Bite time	2019.4.16	2019.6.17
Day1	1, For half an hour, he found it difficult to breathe 2, Four hours later, he complained of shortness of breath, dizziness and difficulty lifting his upper limbs. 3, The patient was administered *Bungarus multicinctus* antivenom.	1, The left hand was noticeably swollen and had limited movement, subcutaneous congestion, swelling around the skin, high skin temperature, and tenderness in the left hand and forearm. 2,6,000 IU of antivenom serum was administered (*Agkistrodon halys* antivenom is a monovalent antivenom)
Day3	symptoms gradually subsided,	After half a month of conservative treatment, the swelling of the patient's left hand improved, with only mild tenderness and no other symptoms.
Day5	Leave hospital	–
Day9	–	The hand is still swollen and tender
Day15	–	there was local blackening and tissue necrosis
Day16	–	1, Local open decompression and thorough debridement were performed 2, Postoperative vacuum sealing drainage (VSD) negative pressure suction was performed 3, skin grafting
Day27	–	The wound eventually healed, and the patient was discharged

**Fig. 2:**
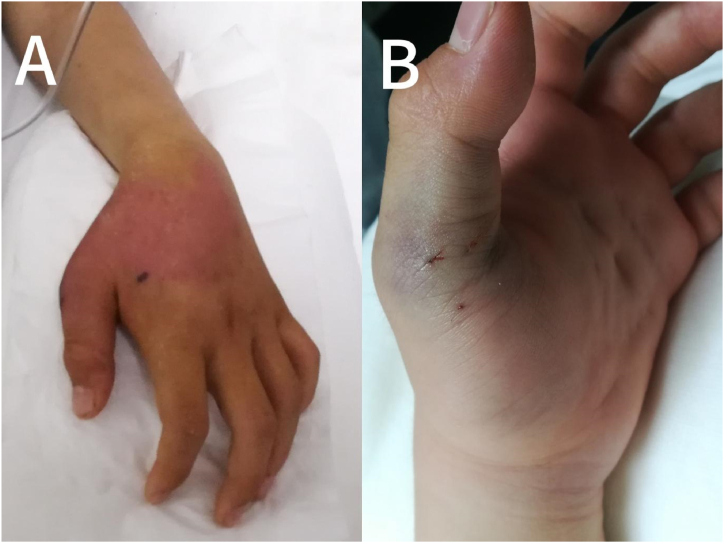
A,The pit viper had bitten the boy on the left hand, which was visibly swollen and ecchymotic; B,local blackening and tissue necrosis.

**Fig. 3:**
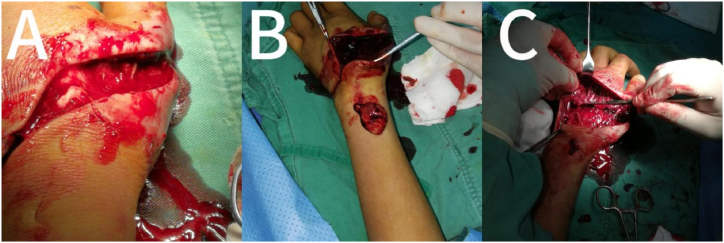
The boy underwent partial decompression and thorough debridement on his left hand.

**Fig. 4:**
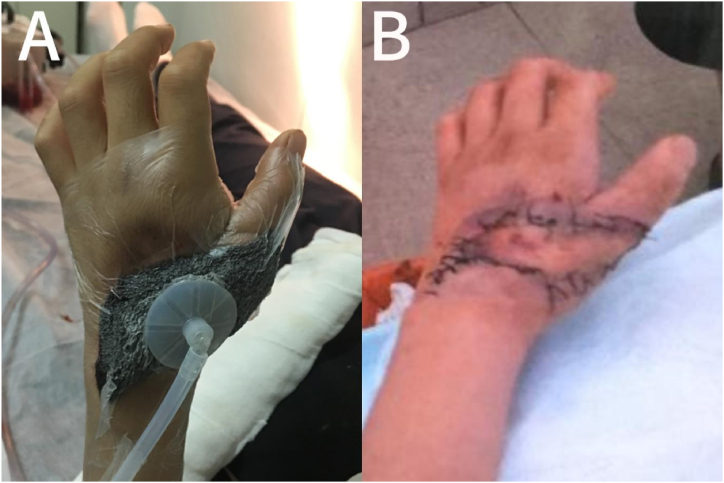
A,Postoperative VSD negative pressure suction was performed after the operation; B,The wound has healed well after the operation.

Approximately two months later, on June 17, 2019, the boy was bitten by a venomous snake again, this time by a short-tailed pit viper *(Gloydius brevicaudus)* (Fig. 1B). The viper, also purchased online, is a *short-tailed pit viper* from Zhejiang province in South China. This snake's venom is a combination of blood and muscle toxins. In this case, it was the left hand that was bitten. Physical examination results were as follows: temperature, 37 °C; pulse rate, 70 beats/min; breathing rate, 17 beats/min; blood pressure, 135/85 mmHg; and SpO_2_, 98 %. The patient was conscious. Bilateral pupils were equally enlarged, homologous, and sensitive to light. Breathing was clear, with no dry and wet rales. Heart rate was 70 times/min with a regular rhythm. The abdomen was flat and soft, with no tenderness and rebound pain. The liver and spleen were not palpable. The left hand was noticeably swollen and had limited movement, subcutaneous congestion, swelling around the skin (Fig. 2A), high skin temperature, and tenderness in the left hand and forearm. There were no abnormalities in the routine blood tests, liver and kidney function, and coagulation series examination on admission. Immediately after admission, 6000 IU of antivenom serum was administered (*Agkistrodon halys antivenom* is a monovalent antivenom), tetanus immunoglobulin was routinely administered, and no allergic reaction or serum sickness type reactions occurred. The patient received a combination of early glucocorticoid administration (dexamethasone 10 mg/d, intravenous drip, for three days), infection control (Flucloxacillin), fluid and electrolyte maintenance, and acid-base balance. The patient was administered Jidesheng snake tablets(The initial oral dosage is 20 tablets (8g), followed by 10 tablets every 6 hours) [8], a traditional Chinese medicine used in China to treat snake wounds, administered orally and topically, simultaneously with a local wet compress of magnesium sulfate. These were performed aseptically in the hospital. A few days later, the swelling of the patient's left hand improved, with only mild tenderness and no other symptoms. After half a month of conservative medical treatment, the boy's left hand showed no signs of remission, and there was local blackening and tissue necrosis (Fig. 2B). Local open decompression and thorough debridement were performed (Fig. 3A-C). Postoperative vacuum sealing drainage (VSD) negative pressure suction was performed (Fig. 4A). The surgeons then removed a piece of skin from the boy's left groin and grafted it onto the wound on his left hand. The wound eventually healed, and the patient was discharged one month later (Fig. 4B). The temporal sequence of the second bite is shown in Table 1.

## 3 Discussion

According to the effects of snake venom on the body, venomous vipers can be divided into neurotoxic, hematotoxic, cytotoxic, and hybrid venomous snakes [6]. In China, the most common neurotoxic snakes are *B. multicinctus* and *sea snakes*. The most common ematotoxic snakes are mainly *Trimeresurus stejnegeri*, *Protobothrops jerdonii*, and *adders*. *Cytotoxic snakes* are mainly cobras [9]. Mixed venomous snakes include *king cobras*, *vipers*, and *five-step snakes (agkistrodon)* [8]. In snakebite cases, pictures of the snake can help identify the type of snake and therefore the type of venom [10]. In our case, the photos provided by the patient's father were instrumental in determining the type of snake that bit the boy.

The mortality rate of *B. multicinctus* bite envenomation ranks first among venomous snake bite poisoning in China [11]. Neurotoxins are divided into β-neurotoxin (β-NT) and α-neurotoxin (α-NT). These act on acetylcholine receptors in motor nerve endings (presynaptic) and motor endplates (postsynaptic), respectively, both of which block normal nerve conduction and cause neuromuscular flacalgia [12,13]. The main cause of death is acute respiratory failure caused by neurotoxicity. It is characterized by small localized wounds and mild bites. Early clinical manifestations include eyelid drooping, dysphagia, respiratory paralysis, respiratory failure, and even respiratory arrest [14] The short-tailed pit vipers (*Gloydius brevicaudus*) are primarily toxic to blood circulation and muscle. In severe cases, it can lead to coagulation dysfunction and localized muscle necrosis [15]. It is characterized by obvious localized wounds, bleeding, limb swelling, severe pain, and sometimes secondary tissue necrosis and infection. There are many types of toxins. For example, serpentine protease acts on the vascular wall, damaging related structures of the vascular wall, inducing the release of bradykinin, etc, and leading to bleeding. The hemolytic factor of snake venom can act directly on the blood cell membrane, increasing its permeability and brittleness [16]. Phospholipases A2, metalloproteinases activate pro-inflammatory mechanisms that cause edema, blister formation, and local tissue necrosis. The patient developed local necrosis after the second venomous snake bite. Studies have shown that in addition to the direct destruction of venom toxins, inflammation, oxidative stress, and secondary wound infection caused by snake bites can lead to local tissue necrosis, which should be addressed during treatment [17,18]. This suggests that we should prevent rare complications during treatment, such as bilateral adrenal and pituitary bleeding after a Russell venomous snake bite [19].

Antivenom is the only specific treatment for snakebite envenoming. Antivenom immunoglobulins (antivenoms) are immunoglobulin or immunoglobulin fragments extracted from the plasma of animals (horse or sheep) immunized against one or more snake venom [[20], [21], [22]]. The monovalent antivenoms on the market in China mainly include anti-agkistrodon, anti-silver ring, anti-cobra, and anti-five-step snake antivenoms. Antivenoms are xenogeneic and can cause anaphylactic or seropathic reactions [23], such as rash, fever, tachycardia, nausea, and anaphylactic shock. Therefore, the clinical manifestations and laboratory results of patients should be closely observed after the administration of antivenoms. Due to the nature of the sites (puncture wounds) and the fact that snakes carry pathogenic organisms, including clostridium tetani as part of their normal oral microflora [24], Tetanus antitoxin (TAT) or horse tetanus immunoglobulin should be routinely used after snake bites; however, the use of antivenom should be avoided during the tetanus skin test. Antivenoms should be started 1h after skin testing and medication to avoid overlapping allergies or adverse reactions. Early use of glucocorticoids can reduce inflammation, hemolysis, allergic reactions caused by snake venom, capillary permeability, and local swelling and bleeding. Small-scale clinical studies have shown that local negative VSD has a good effect on swelling, decay, and even necrosis of the affected limb, which is helpful in preventing the occurrence of compartment syndrome [25]. Jidesheng snake tablets are traditional Chinese herbal medicine. The main ingredients of Jidesheng snake tablets are Gan chan (Succys Bufo), Dijincao (Herba Euphorbiae Humifusae), Chonglou (Rhizoma Paridis Chonglou) and Wu gong (Scolopendra). Ganchan (Succys Bufo) can relieve pain, acute inflammation, and edema. Dijincao can regulate cellular immunity and delay apoptosis. Jidesheng tablets have antipyretic, detoxification, anti-inflammatory, analgesic, and other effects [26]. In China, they are widely used in clinical practice. Research shows that Jidesheng snake tablets have good effects on swelling and pain after venomous snake bite [27].

We conducted a relatively in-depth investigation as to why the boy repeatedly purchased venomous snakes online. It turns out that the boy's parents are divorced; he lives with his grandparents and does not usually communicate with other children. It is likely that, due to the absence of his mother and the current family situation, he developed psychological problems. He is solitary and likes to keep snakes as pets to relieve stress. He often buys pet snakes online through his father's mobile phone. After he was discharged, we arranged for him to see a psychiatrist, and he no longer feeds venomous pets. Recently, venomous snakes previously confined to southern China, such as the *B. multicinctus* and the *cobra*, have also emerged in northern China, mainly bought by pet lovers through online shopping. The availability of venomous snakes contradicts the availability of antivenoms, which makes it difficult to get prompt treatment after bites. In addition, in China, *B. multicinctus* and *Gloydius brevicaudus* are protected animals, which are extremely toxic and are prohibited from selling online, but minors in this case can easily buy them through online shopping, which suggests relevant departments should strengthen the management of venomous animals with wildlife protection laws, improve the supervision level of pet sales platforms, strengthen the protection of minors, carry out the propaganda of snakebite prevention knowledge, and enable pet breeders to master the relevant treatment ability.

## 4 Conclusion

In conclusion, prompt identification of the snake is essential for determining its venomous nature in order to minimize toxin absorption or counteract absorbed toxins, and the corresponding antivenoms should be used as soon as possible according to the species of venomous snakes to avoid rare complications. With the ease of transporting non-local snakes through online shopping and occurrences of snake bites in non-snake habitats, it is imperative for regional hospitals to increase their supply of anti-snake serum to avoid treatment delays. Relevant government departments should enhance supervision measures to prevent such incidents resulting from online shopping.

## CRediT authorship contribution statement

**Ming Gao:** Writing – original draft, Methodology, Investigation. **Xiangxing Zhang:** Writing – original draft, Investigation, Formal analysis, Data curation, Conceptualization. **Tianzi Jian:** Writing – original draft, Validation, Data curation. **Cece Sun:** Resources, Investigation, Formal analysis, Conceptualization. **Guangcai Yu:** Supervision, Software, Project administration, Methodology, Conceptualization. **Yikai Gao:** Writing – original draft, Project administration, Investigation, Formal analysis. **Baotian Kan:** Writing – review & editing, Supervision, Conceptualization. **Xiangdong Jian:** Writing – review & editing, Supervision, Funding acquisition, Formal analysis, Data curation, Conceptualization.

## Ethical approval

Ethical approval for this study was obtained from Shandong University Qilu Hospital of Ethics Committee (KYLL-2019-296).

## Data availability statement

The original data presented in the study are included in the article, further inquiries can be directed to the corresponding authors.

## Ethics declarations

This study was approved by the Ethics Committee of Qilu Hospital of Shandong University, and informed assent was obtained from patient in addition to consent from his father.Informed consent has been obtained from all individuals included in this case report/case series. The individuals providing consent understand that the publication of clinical conditions, research findings, de-identified images, clinical reports, and any other information related to each patient discussed in the journal will be widely and freely accessible to the public, without restrictions. The signed informed consent acknowledges that the publication may be promoted on the journal's website, possibly featured in news and/or social media, and may be utilized for future research advancements.The privacy and confidentiality of the patients involved have been carefully considered. Any personal information that could potentially identify the patients has been appropriately de-identified to protect their privacy. The patients have been made aware that their information may be used for academic research, medical publications, and may be disseminated in various public domains.This submission adheres to the ethical standards and guidelines set forth by Heliyon regarding the publication of case reports and case series. The authors affirm that they have complied with all relevant ethical considerations, and this submission does not violate any ethical or legal standards.

## Declaration of competing interest

The authors declare that they have no known competing financial interests or personal relationships that could have appeared to influence the work reported in this paper.
